# A comparative study of ultrasound-guided percutaneous nephrolithotripsy and x-ray-guided percutaneous nephrolithotripsy in the treatment of complex renal calculi without hydronephrosis

**DOI:** 10.12669/pjms.38.8.5904

**Published:** 2022

**Authors:** Shao-wei Xue, Ming-bo Zhang, Yu-kun Luo, Nan Li, Shang-yi Shi

**Affiliations:** 1 Shao-wei Xue, Department of Ultrasound Diagnosis, The First Medical Center of Chinese PLA General Hospital, Beijing 100853, P. R. China; 2 Ming-bo Zhang, Department of Ultrasound Diagnosis, The First Medical Center of Chinese PLA General Hospital, Beijing 100853, P. R. China; 3 Yu-kun Luo, Department of Ultrasound Diagnosis, The First Medical Center of Chinese PLA General Hospital, Beijing 100853, P. R. China; 4 Nan Li, Department of Ultrasound Diagnosis, The First Medical Center of Chinese PLA General Hospital, Beijing 100853, P. R. China; 5 Shang-yi Shi Department of Urology, Beijing Jingmei Group General Hospital, 102399 Beijing, China

**Keywords:** Ultrasound guidance, X-ray guidance, Percutaneous nephrolithotomy, Complex renal calculi without hydronephrosis

## Abstract

**Objective:**

To compare and analyze the clinical effects of ultrasound-guided percutaneous nephrolithotripsy and X-ray-guided percutaneous nephrolithotripsy in the treatment of complex renal calculi without hydronephrosis.

**Methods:**

Eighty patients with multiple stones without hydronephrosis were admitted at Department of Ultrasound Diagnosis, The first medical center of Chinese PLA General Hospital from January 21, 2020 to December 21, 2020 randomly divided: into two groups: experimental group and control group, with 40 cases in each group. Patients in the experimental group were treated with ultrasound-guided percutaneous nephrolithotomy, while those in the control group were treated with X-ray-guided percutaneous nephrolithotomy. The differences in operation time, channel establishment time, channel number, blood loss and stone clearance rate between the two groups were compared and analyzed. Venous blood was drawn before surgery and on the first day after surgery, and serum creatinine, urea nitrogen, blood β2-microglobulin, blood uric acid and other renal indexes were detected. Moreover, renal parenchymal injury was compared between the two groups by renal static imaging, and the incidence of postoperative complications such as pain, fever, urination through incision and injury of surrounding organs were compared and analyzed.

**Results:**

The operation time, channel establishment time, channel number and blood loss in the experimental group were significantly lower than those in the control group, with statistically significant differences (p<0.05). The postoperative renal injury score of the experimental group was 1.03±0.37, which was lower than 1.85±0.63 of the control group (p=0.00); Postoperative Cr, BUN, blood β 2-microglobulin and other indicators in the control group were significantly higher than those in the experimental group, with statistically significant differences (p<0.05). The incidence of peripheral organ injury in experimental group was lower than that in control group, with a statistically significant difference (p=0.04).

**Conclusion:**

Ultrasound-guided percutaneous nephrolithotomy is a safe and effective treatment regimen, boasting various advantages such as real-time monitoring of the surgical process, more accurate and clear channel establishment, avoidance of large vessel injury, shortening of surgical time, alleviation of kidney injury and reduction of surgical complications, which is more advantageous for the treatment of complex renal calculi.

## INTRODUCTION

In the wake of change of people’s eating habits, complex renal calculi are becoming more common in clinical practice.^1^ Complex renal calculi generally refer to staghorn stones of the kidney, and also include multiple renal calculi and renal calculi with special anatomical structure, such as horseshoe renal calculi.^2^ Once renal calculi occur, the impact on the affected kidney increases dramatically. In order to protect the renal function to the greatest extent, the calculi should be completely removed during operation. However, complex renal calculi are difficult to handle surgically due to their large size, large calculus load, or abnormal anatomical structures in the kidney. For this reason, the treatment of complex renal calculi is a clinically recognized technical barrier. Percutaneous nephrolithotripsy (PCNL) is the gold standard for the treatment of complex renal calculi and is widely applied in clinical practice, boasting advantages of small trauma, high lithotripsy clearance rate and low postoperative complications.^3^

The key of PCNL is the establishment of a percutaneous renal channel, which have the advantages of less bleeding, high calculus clearance rate and less complications. For the present, the preferred methods for establishing PCNL channels include C-arm X-ray positioning and ultrasonic positioning. For patients with hydronephrosis, the above channel establishment method is relatively easy to operate and has a high success rate. while for those with complex calculi without hydronephrosis, they have difficulty in establishing the channel due to the large calculus volume, inconspicuous hydronephrosis and relatively small intra-renal space.

In this paper, a comparative study was conducted on ultrasound-guided percutaneous nephrolithotripsy and X-ray-guided percutaneous nephrolithotripsy in the treatment of patients with complex renal stones without hydronephrosis, and the former was considered to have certain advantages.

## METHODS

Eighty patients with multiple stones without hydronephrosis were admitted at Department of Ultrasound Diagnosis, The first medical center of Chinese PLA General Hospital from January 21, 2020 to December 21, 2020 randomly divided into two groups: experimental group and control group, with 40 cases in each group. The study was approved by the Institutional Ethics Committee of The first medical center of Chinese PLA General Hospital (Number:S2019-178-01; Date:July 13, 2019), and written informed consent was obtained from all participants.

### Inclusion criteria:

- Patients< 75 years old;
- Patients with multiple calculi in the renal pelvis in preoperative ultrasound, intravenous pyelography, and urinary system CT imaging;^4^
- Patients without obvious hydronephrosis;
- Patients with calculus diameter >3 cm;
- Patients who can cooperate to complete the study;
- Patients who volunteered to participate in the study and signed the consent form.

### Exclusion criteria:

- Patients with severe hydronephrosis and abnormal renal function;
- Patients with severe urinary tract infection and unsatisfactory control;
- Patients with a history of kidney surgery;
- Patients with abnormal coagulation function;
- Patients with cardiopulmonary dysfunction who cannot bear surgery;
- Patients with underlying diseases that cannot be satisfactorily controlled, such as diabetes and hypertension;
- Patients who cannot cooperate to complete the study.

Patients in the experimental group underwent ultrasound-guided percutaneous nephrolithotomy, while those in the control group underwent X-ray-guided percutaneous nephrolithotomy. There were 22 males and 18 females in the experimental group, aged from 47 to 75 years, with an average of 63.31±12.38 years; There were 25 males and 15 females in the control group, aged from 50 to 72 years old, with an average of 63.27±11.85 years old. No significant difference can be seen in the comparison of general data between the two groups, which was comparable (Table-I).

**Table-I:** Comparative analysis of general data between the two groups (X̄ ±S) n=40.

Indicators	Experimental group	Control group	t/χ^2^	p
Male (%)	22	25	0.46	0.50
Age (years old)	63.31±12.38	63.27±11.85	0.01	0.98
Calculus site			0.20	0.65
Left kidney (%)	21	23		
Right kidney (%)	19	17		
Calculus volume (cm^2^)	4.43±1.20	4.12±1.03	1.24	0.22
Calculus nature			0.49	0.49
Multiple calculus	27	24		
Staghorn calculus	13	16		
Associated symptoms				
Pain (%)	11	12	0.06	0.81
Hematuria (%)	7	6	0.09	0.76
Urinary tract infection (%)	9	8	0.07	0.79
No symptoms (%)	13	14	0.06	0.81

p>0.05.

### Surgical methods:

General anesthesia was performed in both groups. First, the ureteroscope was inserted into the bladder through the urethra in the lithotomy position, and the ureteral catheter was inserted into the ureter along the ureteroscope. In case of bilateral simultaneous surgery, bilateral ureteral catheters were indwelled, and the ureteral catheters were connected to a pressurized flushing system to establish artificial hydronephrosis. Subsequently, the patients were changed to a prone position with their waist raised. In the experimental group, ultrasound scan of the target kidney was performed, and the puncture needle was inserted into the middle calyx of the target kidney under the guidance of B-mode ultrasound. When the needle core was withdrawn, urine was found to flow out, the special guide wire was inserted, the needle sheath was removed, the skin was cut with a sharp knife, and the needle passages were successively expanded with fascial expander along the guide wire to the F16 fascial expander. At the same time, a peel-Away thin skin sheath was inserted, F16 fascial dilator was pulled out, and then a metal dilator was inserted to expand the original fistula to F24. During the expansion of the channel, an ultrasound probe was used to monitor in real time to ensure that the expansion is in place. The F24 sheath was inserted and the ultrasonic gravel probe was inserted to break the visible stones in the field of vision and absorb them synchronously. For calculi outside the blind area of the standard channel visual field, a puncture point was selected again, and dual channels or multiple channels were established under ultrasound guidance for lithotripsy and stone removal. After the calculus clearance effect is satisfactory, the F5 ureteral stent tube (DJ tube) was indwelled, the nephroscope was withdrawn, and the nephrostomy tube was placed and fixed in the collection system. In the control group, a calculus extraction channel was designed according to preoperative imaging data. A puncture needle was placed on the body surface to simulate the projection of the puncture path on the back skin, and a clamp was placed on the body surface projection point of the renal pelvis. Ioprolamine was injected through the ureteral catheter to develop the collection system under X-ray. After the instantaneous C-arm X-ray machine was positioned, the puncture angle and depth were determined, the puncture needle was inserted to the corresponding depth, and the needle core was removed at the right time. At this time, urine was found to flow out, guide wire was inserted, and the lithotripsy process is the same as that of the experimental group. Repeat the process if multiple channels are required for lithotripsy.

### Observation Indicators

***Surgery-related indicators:*** The differences in the operation time, channel establishment time, channel number, bleeding volume, calculus clearance rate and other indicators between the two groups were compared and analyzed;

***Renal injury-related indicators:*** Venous blood was drawn from the two groups before and first day after surgery to detect blood creatinine, blood urea nitrogen, blood β2-microglobulin, blood uric acid and other renal function indicators. The renal static imaging technique was used to compare and analyze the renal parenchymal damage of the two groups postoperatively. Method for judging the results of the radionuclide renal static imaging: two experienced nuclear medicine physicians read the radiographs together. The area of interest (ROI) technique was used to delineated the plane area of both kidneys and the area of sparse or defective areas, and the ratio of the damaged area to the area of both kidneys was calculated. According to the renal injury scoring criteria in the literature,^6^ the degree of renal injury was divided into five grades: 0 point means no damage, with an area ratio of 0%; one point mean uncertain or mild injury, with an area ratio of <5%. Two point indicate mild injury with an area ratio of 5-10%, three points represent moderate injury with with an area ratio of 10-30%, four points indicate severe renal parenchymal injury, with an area ratio of>30%; The incidence of postoperative complications such as pain, fever, oozing from the incision, and peripheral organ injury between the two groups was comparatively analyzed.

### Statistical Analysis:

All the data were statistically analyzed by SPSS 20.0 software, and the measurement data were expressed as (X̄± s). Two independent sample T tests were used for data analysis between the experimental group and the control group. Paired T test was used for data analysis between the two groups before and after treatment, and χ^2^ test was used for rate comparison. P< 0.05 indicates a statistically significant difference.

## RESULTS

Operation time, channel establishment time, channel number, bleeding volume, calculus clearance rate: The comparative analysis of surgical data between the two groups is shown in Table-II. The operation time, channel establishment time, number of channels, and bleeding volume of the experimental group were significantly lower than those of the control group, with statistically significant differences (p<0.05); no statistically significant difference can be seen in the calculus clearance rate between the two groups (p=0.40).

**Table II:** Comparative analysis of surgical data of the two groups (X̄ ±S) n=40.

Group	Operation time (min)*	Channel establishment time (min)*	Bleeding volume (ml)*	Calculus clearance rate (%)	Number of channels*
Experimental group	78.61±15.42	11.36±3.75	67.48±8.42	38(95%)	2.15±0.42
Control group	86.96±16.70	16.27±5.39	83.55±11.03	36(90%)	3.47±0.36
t/χ2	2.32	4.72	7.42	0.72	15.10
p	0.02	0.00	0.00	0.40	0.00

* p<0.05.

The renal injury score was 1.03±0.37 in the experimental group and 1.85±0.63 in the control group, indicating that the degree of kidney injury in the experimental group was lower than that in the control group (p=0.00). Renal function examination showed that postoperative Cr, BUN, blood β2-microglobulin and other indicators in the control group were significantly higher than those in the experimental group, with statistically significant differences(p<0.05), while postoperative uric acid level showed no significant difference between the two groups (p=0.81) (Table-III).

**Table-III:** Comparative analysis of renal function index scores between the two groups (X̄ ±S) n=40.

Indicators	Observation point	Experimental group	Control group	t	p
Cr (mmol/L)	Before treatment	73.55±8.37	73.46±7.95	0.05	0.96
	After treatment*	84.70±6.38	89.74±9.26	2.83	0.01
BUN (mmol/L)	Before treatment	5.37±1.25	5.40±1.54	0.10	0.92
	After treatment*	7.45±1.30	11.33±3.21	7.08	0.00
β2-microglobulin (mg/L)	Before treatment	3.46±0.43	3.42±0.39	0.44	0.67
	After treatment*	5.63±0.32	7.37±0.46	19.64	0.00
Blood uric acid (μmol/L)	Before treatment	323.26±23.67	325.40±23.58	0.41	0.68
	After treatment	324.55±21.47	325.74±23.66	0.24	0.81

* p<0.05

In the experimental group, there were 10 patients (25%) with postoperative complications, all of which were mild (Clavien I-II), which were improved after conservative treatment or drug treatment, and no moderate or severe complications such as organ injury occurred. In the control group, there were 19 postoperative complications (40%), of which 17 were mild, including two patients with pleural stimulation, which were improved after conservative treatment or drug treatment, and two were moderate, including one case of pleural injury and one case of colon injury, which were improved after surgical intervention. There was significant difference in the incidence of organ injury between the two groups (p=0.04) (Table-IV), but there was no significant difference in the incidence of other complications (p>0.05).

**Table-IV:** Comparative analysis of the incidence of surgical complications between the two groups (X̄ ±S) n=30

Indicators	Postoperative pain	Fever	Oozing from the incision	Organ damage *
Experimental group	5	3	2	0
Control group	7	5	3	4
χ^2^	0.39	0.56	3.13	4.21
p	0.53	0.45	0.07	0.04

* p<0.05

## DISCUSSION

The continuous development of surgical techniques and instruments, the majority of current stone surgeries are minimally invasive due to the natural cavity of the urinary system.^7^ Various minimally invasive methods such as PCNL, extracorporeal shock wave lithotripsy, ureteroscopic lithotripsy, and soft ureteroscopic lithotripsy are preferred for the treatment of renal calculi. However, for complex calculi such as cast renal calculi, multiple calculi and staghorn calculi, PCNL, a lithotriptic clearing technology for calculi in the collecting system and on the ureter via the percutaneous renal channel,^8^ is still indispensable.^9^

The most critical step affecting the efficacy of PCNL surgery is the establishment of working channels.^10^ Unreasonable percutaneous renal channels increase surgical risks, such as bleeding, kidney injury, and infection.^11^ For complex renal calculi, percutaneous renal channels are required to pass as much as possible through the target calyx into the renal pelvis to clear the calculi. Meanwhile, excessive swing of the nephroscope in the kidney during the operation should be avoided to cause caliceal neck fracture and bleeding, which would affect the operating field of vision, and damage of adjacent tissues and organs should be avoided to reduce intraoperative and postoperative complications and shorten the operation time. In view of this, precise design and establishment of a reasonable PCNL working channel is the key to successful surgery.^12^ The establishment of the best lithotripsy channel depends on a satisfactory surgical position and the way of guiding positioning. Currently, X-ray guidance and ultrasound guidance are commonly used puncture guidance methods in clinical practice.^13^ Both have their own unique advantages. Specifically, X-ray guidance is characterized by accurate positioning, clear images, and simple operation. X-ray fluoroscopy is close to preoperative angiography, under which the calculi can be easily identified by the surgeon, and the overall picture of guidewire, stones and fistula can be intuitively displayed. However, X-ray also has many disadvantages: radiation damage, no real-time monitoring, only contrast enhancement can be injected into the collection system to show the operation field, and unsatisfactory soft tissue development. It is, therefore, impossible to protect the large blood vessels during the operation and avoid damage to adjacent organs.^14^ In addition, X-ray provides mostly two-dimensional plane images, but less spatial and three-dimensional information about renal aggregation system and stone location, etc., and the anterior calyces and posterior calyces can also overlap and interfere with each other.^15^

In contrast, ultrasound-guided positioning has its greatest advantages in terms of low radiation damage to patients and medical staff, no special protection or radiological diagnosis and treatment equipment required, convenient operation, and short puncture time.^16^ It can clearly show the calyces and calculi, water accumulation in each group of kidney, effectively avoid ribs, large blood vessels, etc., and monitor the depth and angle of needle insertion in real time.^17^ For patients with unobvious hydronephrosis, vision can be clearly and satisfactorily established after intraoperative hydronephrosis.^18^ Ultrasound can clearly display the anatomical relationship between the pleura, lung, colon, liver, spleen and other substantial organs and kidney, avoiding the surrounding important organs.^19^ The doppler function of ultrasound can clearly display the large blood vessels in the kidney, so that the needle can be inserted from the avascular plane of the kidney, which reduces the intraoperative and postoperative bleeding. It can be seen that ultrasound boasts advantages such as clear visual field, shortened operation time, improved calculus clearance rate, and reduced probability of rescheduling surgery due to visual field bleeding.^20^ During the operation, ultrasound exploration can be used to find out the effect of calculus clearance and whether calculi remain, and the second or third channel can be established by puncture again according to the position of the stone. Ultrasound-guided PCNL has the advantages of higher success rate of single needle puncture, fewer puncture times and shorter puncture time.^21^ Lazarus et al.^22^ believed that ultrasound guided puncture could significantly reduce the puncture time and the number of puncture statistically.

It was confirmed in our study that compared with X-ray guidance, ultrasound-guided PCNL significantly reduced the operation time, channel establishment time, channel number and blood loss (p<0.05). The renal injury score, Cr, BUN and blood β2-microglobulin in the experimental group were lower than those in the control group (p=0.00), and the incidence of peripheral organ injury was lower than that in the control group, with a statistical significance (p=0.04).

### Limitations of the study:

Small sample size was included, patients were not systematically followed up, and patients with anatomic abnormalities such as horseshoe kidneys, ectopic kidneys and other complex calculi were not included in the observation. In view of this, effective countermeasures are being taken to actively expand the sample size, further increase the content of follow-up, and include the above-mentioned patients with anatomical abnormalities for systematic research, in order to more objectively and systematically evaluate the advantages of ultrasound-guided channel establishment.

## CONCLUSION

Ultrasound-guided percutaneous nephrolithotomy is a safe and effective treatment regimen in the treatment of complex renal calculi without hydronephrosis, boasting various advantages such as more accurate and clear channel establishment, avoidance of large vessel injury, shortening of surgical time, and reduction of surgical complications.

### Authors’ Contributions:

**SWX &** **MBZ:** Designed this study and prepared this manuscript, and are responsible and accountable for the accuracy or integrity of the work.

**YKL & NL:** Collected and analyzed clinical data.

**SYS:** Significantly revised this manuscript.
